# *Argostemma baotingense* (Rubiaceae), a new species from Hainan Island, South China

**DOI:** 10.3897/phytokeys.276.195404

**Published:** 2026-06-18

**Authors:** Shu-Peng Dong, Lang-Xing Yuan, Qing-Long Wang, Hu-Biao Yang

**Affiliations:** 1 Tropical Crops Genetic Resources Institute, Chinese Academy of Tropical Agricultural Sciences, Danzhou, Hainan 571737, China Tropical Crops Genetic Resources Institute, Chinese Academy of Tropical Agricultural Sciences Hainan China

**Keywords:** *
Argostemma
*, new species, taxonomy

## Abstract

*Argostemma baotingense*, a new species of Rubiaceae from Hainan Island, southern China, is described. This new species resembles *A. bachmaense* but can be distinguished by its suberect or creeping stem, linear leaves with a greenish-white abaxial surface, white and glabrous inflorescence, and glabrous calyx. An identification key and photographs of the nine *Argostemma* species in China are provided.

## Introduction

The genus *Argostemma* Wallich (Rubiaceae) is an herbaceous genus containing 181 species (POWO 2026), mostly distributed in southern, eastern, and southeastern Asia, with only two species endemic to west tropical Africa (Chen and Taylor 2011). *Argostemma* species are mainly lithophytes or occasionally epiphytes and prefer to grow in shady areas with humid conditions, near streams, waterfalls, and wet riverbeds with moss-laden rocks (Sridith and Puff 2000). The genus is characterized by opposite or verticillate leaves, white and rotate corollas, a glabrous inner surface of the corolla tube, free anthers or anthers coherent into a tube, and anthers opening by longitudinal slits or apical pores (Puff et al. 1995; Chen and Taylor 2011; Liu et al. 2022). The diversity centers of *Argostemma* are in the Malay Peninsula (50 spp.), Thailand (31 spp.), and Borneo (28 spp.) (Ridley 1923; Bremer 1989; Sridith 1999, 2007). In China, eight species of *Argostemma* were recorded, with distributions in Guangdong, Hainan, Yunnan, Guangxi, and Taiwan (Chen and Taylor 2011; Liu et al. 2022; Huang et al. 2024).

During a field survey conducted in March 2025, an unknown *Argostemma* species was found in Tonganling Mountain, Hainan Island, southern China. The species has a suberect or creeping stem, linear leaves with a greenish-white abaxial surface, white and glabrous inflorescence, and a glabrous calyx, which distinguish it from other known *Argostemma* taxa. After carefully consulting herbarium specimens and relevant literature on *Argostemma*, the species was concluded to be new and is described herein (Bremer 1989; Sridith 1999, 2007, 2012; Sridith and Larsen 2004; Puff 2009; Tanaka et al. 2010; Choudhary et al. 2013; Lanorsavanh and Chantaranothai 2016, 2018, 2019; Nuraliev et al. 2017; Quang et al. 2019; Hsu et al. 2020; Lanorsavanh et al. 2020; Truong et al. 2020; Vu et al. 2020; Balan et al. 2021; Fang et al. 2022; Sadokpam et al. 2023; Blasco et al. 2024; Huang et al. 2024; Lalhlupuii et al. 2025; Quan et al. 2025).

## Materials and methods

Specimens of the new species were collected from Tonganling Mountain, Hainan Province, and living plants were introduced to the nursery of the Tropical Crops Genetic Resources Institute of the Chinese Academy of Tropical Agricultural Sciences. Type specimens were deposited in the herbaria of the Tropical Crops Genetic Resources Institute of the Chinese Academy of Tropical Agricultural Sciences (ATCH) and South China Botanical Garden (IBSC). Habitat information was recorded during the field surveys. Morphological observations and measurements were carried out on living plants both *in situ* and *ex situ*.

## Taxonomic treatment

### Argostemma baotingense

Taxon classificationPlantaeGentianalesRubiaceae

S.P.Dong & H.B.Yang, sp. nov.

7A73B8A4-DEC5-55FB-907D-747D545CAA7F

urn:lsid:ipni.org:names:77381817-1

Fig. 1

#### Type.

China. Hainan: Baoting County, Tonganling Mountain, on the rocks of the valley in the forest, 18.55941219, 109.54065926, alt. 730 m, 30 May 2025, *Shu-Peng Dong*, *Jian-Qi Dong*, *Long-Wei Pang DSP202503* (holotype: ATCH, isotype: IBSC1104113, 1104114).

**Figure 1.**
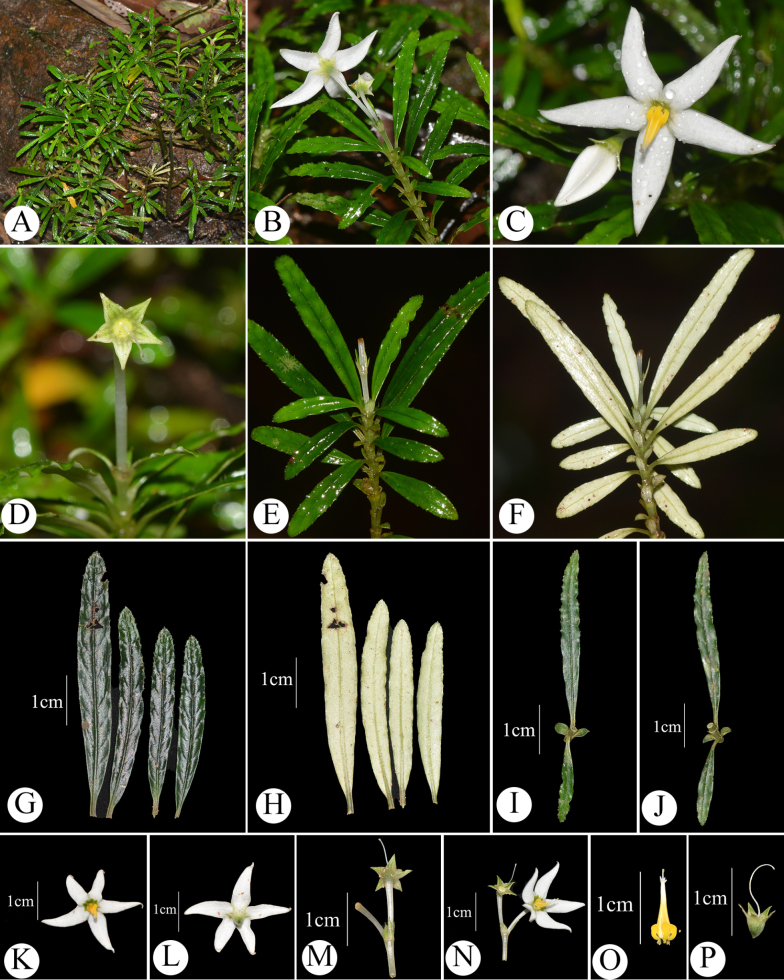
*Argostemma baotingense*. **A**. Plant in natural habitat; **B**. Flowering plant; **C**. Flowers; **D**. Calyx; **E**. Leafy branch, adaxial view; **F**. Leafy branch, abaxial view; **G**. Leaves, adaxial view; **H**. Leaves, abaxial view; **I, J**. A node with opposite leaves and stipules; **K**. Flower, apical view; **L**. Flower, bottom view; **M, N**. Inflorescence; **O**. Androecium; **P**. Pistil.

#### Diagnosis.

**Figure 2.**
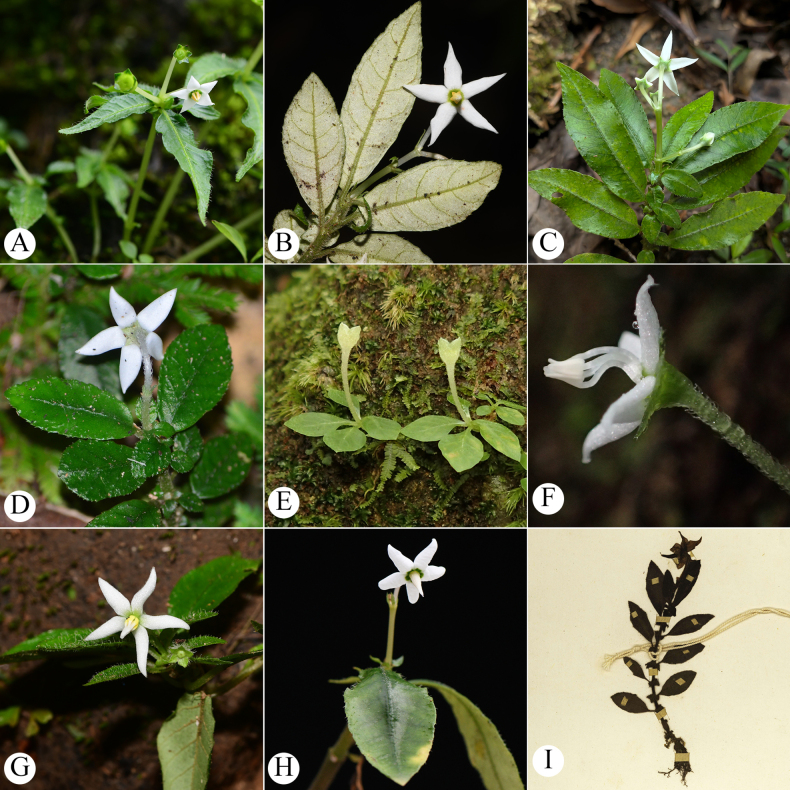
Photographs of seven *Argostemma* species found in China. **A**. *A. verticillatum*; **B, C**. *A. saxatile*; **D**. *A. discolor*; **E, F**. *A. ehuangzhangense*; **G**. *A. solaniflorum*; **H**. *A. yunnanense*; **I**. Holotype of *A. hainanicum*; **A**. Taken by Xin-Xin Zhou from Yunnan; **B**. Taken by Xin-Xin Zhou from Guangdong; **C**. Taken by Yi Huang from Guangdong; **D**. Taken by Si-Yuan Zeng from Hainan; **E, F**. Taken by Yi Huang from Guangdong; **G**. Taken by Xin-Xin Zhou from Taiwan; **H**. Taken by Bu-Yun Zhang from Yunnan; **I**. Provided by You-Pai Zeng and stored in IBSC.

The new species is morphologically similar to *A. bachmaense* T.V.Do but differs by the suberect or creeping stem (vs. erect), the linear leaves (vs. oblanceolate to spatulate), glabrous inflorescence (vs. pubescent), glabrous calyx (vs. pubescent). A detailed morphological comparison between the two species is provided in Table 1. Photographs of seven *Argostemma* species in China have been provided (Fig. 2). A key to all the nine species of *Argostemma* in China is also provided at the end of the text.

**Table 1.** Morphological comparison of *Argostemma baotingense* and *A. bachmaense*.

	*A. baotingense*	*A. bachmaense*
Tuber	absent	globose
Stem	5–15 cm tall, glabrous, suberect or creeping	2.5–5 cm tall, pubescent, erect
Leaves	linear, 1.1–5 × 0.3–0.6 cm	oblanceolate to spatulate, 0.4–2 × 0.15–0.42 cm
Abaxial surface	glabrous, greenish white	pubescent, green
Stipule	broadly ovate, 3–4 × 2–3 mm, glabrous	Broadly ovate to deltoid, 1.2–1.7( 2) × 1.1–1.4 mm, pubescent
Inflorescence	white, 1- or 2-flowered, glabrous	green, 1-flowered, pubescent
Bracts	Four, glabrous, ovate to linear	absent
Sepals	glabrous, light green, triangular	pubescent, dark green, narrowly triangular to lanceolate
Stamens	ca. 8 mm long; filaments yellow	6.5–7.5 mm long; filaments pale green
Style length	ca. 9 mm	8.5–9.5 mm
Stigma	columnar, ca. 0.5 mm long	globose, ca. 0.1 mm long

#### Description.

Lithophytic herbs. Stem 5–15 cm tall, ca. 3 mm in diameter, glabrous, suberect or creeping, internodes 4–9 mm long, terete. Stipules persistent, broadly ovate, 3–4 × 2–3 mm, glabrous, apex acuminate, margin entire. Leaves opposite, slightly anisophyllous, petiole 3–5 mm long, blade linear, 1.1–5 × 0.3–0.6 cm, apex obtuse, margin undulate and strigose, attenuate at base, adaxial surface glossy, dark green, strigose on midrib and sometimes near margins, protuberance on both surfaces, abaxial surface silvery white, glabrous, lateral veins 7–11 pairs, conspicuous adaxially, not visible abaxially. Inflorescence terminal, white, 1- or 2-flowered, peduncle glabrous, ca. 8 mm long, bracts 4, ovate to linear, 3–4 × 1–2 mm, persistent and glabrous, pedicel ca. 11 mm long. Calyx lobes 5, light green, triangular, glabrous, ca. 3 mm long. Corolla white, rotate, glabrous; tube ca. 2 mm long; lobes 5, lanceolate, 10–12 × 4–5 mm, apex acuminate. Stamens 5, ca. 8 mm long; anthers coherent, yellow, dehiscent by longitudinal slits, with connective prolonged, filaments short, ca. 1 mm long, straight, yellow. Ovary inferior, ovules numerous, style ca. 9 mm long, white, stigma columnar, ca. 0.5 mm long, simple, exserted.

#### Phenology.

The new species was observed flowering from May to June.

#### Etymology.

The specific epithet ‘*baotingense*’ is derived from the type locality, Baoting County. The Chinese name is given as ‘保亭雪花’(Pinyin: bǎo tíng xuě huā).

#### Distribution.

**Figure 3.**
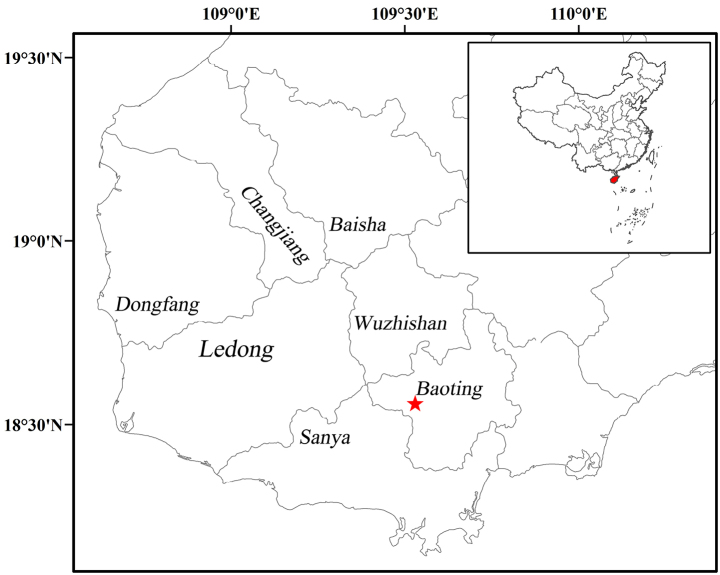
Distribution map of *Argostemma baotingense*.

The new species is known only from the type locality (Fig. 3).

#### Habitat.

It grows on rocks in valleys in the forest. Living plants introduced from the type locality are currently cultivated in the nursery of the Tropical Crops Genetic Resources Institute of the Chinese Academy of Tropical Agricultural Sciences.

#### Conservation status.

Currently, only a single population has been found in the valleys of the Tonganling Mountain forest in Hainan, but its population size is relatively large, and it can reproduce asexually through stems. *Argostemma baotingense* may occur in other areas. As its broader distribution has not yet been fully investigated, it is recommended that it be classified as Data Deficient (DD) under the IUCN Red List criteria (IUCN 2026).

## Discussion

There are two *Argostemma* species distributed in Hainan, *Argostemma discolor* and *Argostemma hainanicum*, both of which are endemic to Hainan. *Argostemma discolor* is widely distributed in Wuzhishan, Diaoluoshan, Bawangling, and Jianfengling; its elliptical leaf shape and hairy calyx make it easy to distinguish from *Argostemma baotingense*. *Argostemma hainanicum* is only distributed in its type locality, Jianfengling. There is little information available, but based on the leaf shape and indumentum of its type specimens, it can also be distinguished from *Argostemma baotingense*.

*Argostemma baotingense* has linear leaves with a greenish-white abaxial surface, glabrous inflorescences, white and glabrous inflorescence, and a glabrous calyx, distinguishing it clearly from *Argostemma* species distributed in China.

*Argostemma baotingense* is similar to *A. bachmaense* T.V.Do but differs by the suberect or creeping stem (vs. erect), linear leaves (vs. oblanceolate to spatulate), glabrous inflorescence (vs. pubescent), and glabrous calyx (vs. pubescent). A detailed morphological comparison between the two species is provided in Table 1.

This species has certain horticultural ornamental value, is suitable for growing in rainforest eco-tanks, and is well loved by plant enthusiasts.

### Key to the nine species of *Argostemma* in China

**Table:** 

1	Tuber globose; leaves verticillate	**2**
–	Tuber absent; leaves opposite	**3**
2	Anthers free, opening by apical pores	***A. verticillatum***
–	Anthers coherent in a cone, opening by longitudinal slits	***A. ehuangzhangense***
3	Leaf blade brownish yellow or pale green abaxially when dry, secondary veins not visible in abaxially	**4**
–	Leaf blade pale white abaxially when dry, secondary veins visible in abaxially	**5**
4	Leaves liner, Leaf blade pale green abaxially when dry	***A. baotingense***
–	Leaves oblong-lanceolate or oblong-ovate, leaf blade brownish yellow abaxially when dry	***A. hainanicum***
5	Calyx glabrous	**6**
–	Calyx pilosulous, strigillose, villosulous, villous, or hirsute	**7**
6	Corolla lobes ovate, ca. 5 mm	***A. saxatile***
–	Corolla lobes lanceolate, 8.5–11 mm	***A. yunnanense***
7	Stem creeping	***A. discolor***
–	Stem suberect or erect	**8**
8	Plant height 2–7(–13) cm; stipule size 1–2 × 1–2 mm; Major leaf size 2.2–4.3 × 0.5–1.4 cm	***A. iriomotense***
–	Plant height (2.5–)5–40 cm; stipule size (2–)3–8 × 1.5–5 mm; Major leaf size (2.5–)4.0–9.7(–11.5) × (l.5–)2.2–3.5 (–3.7) cm	***A. solaniflorum***

## Supplementary Material

XML Treatment for Argostemma baotingense

## Additional information

### Artificial Intelligence (AI) use

The authors accept full responsibility for the content of the manuscript, including the disclosure of any use of AI.

No AI tools were used in the preparation of this manuscript.

### Author ORCIDs

S.-P. Dong https://orcid.org/0009-0004-0409-9551

L.-X. Yuan https://orcid.org/0000-0003-2102-9075

Q.-L. Wang https://orcid.org/0000-0002-9834-6944

H.-B. Yang https://orcid.org/0000-0002-7380-8874

### Data availability

All of the data that support the findings of this study are available in the main text.
